# Altered fecal microbial and metabolic profiles reveal potential mechanisms underlying anemia in patients with chronic renal failure

**DOI:** 10.1128/spectrum.03166-24

**Published:** 2025-06-17

**Authors:** Haichao Wang, Wen Xue, Jiafen Cheng, Yipei He, Yaxiang Song, Dayong Hu, Ai Peng, Changbin Li, Hui Bao

**Affiliations:** 1 Department of Nephrology, Shanghai Tenth People’s Hospital, Tongji University School of Medicine 481875, Shanghai, China; 2 Center for Nephrology and Clinical Metabolomics, Shanghai Tenth People’s Hospital, Tongji University School of Medicine 481875, Shanghai, China; 3 Department of General Practice, Shanghai Tenth People’s Hospital, Tongji University School of Medicine 481875, Shanghai, China; University of Arkansas for Medical Sciences, Little Rock, Arkansas, USA

**Keywords:** chronic renal failure, anemia, gut microbiome, metagenomic, metabolomics

## Abstract

The gut microbiomes communicate with the kidney and may play a crucial role in the development of anemia in patients with chronic renal failure (CRF). However, the alterations in microbiota and their association with functional metabolites remain unclear. We performed metagenomics and untargeted metabolomics in a cohort of 30 patients with anemia of CRF and 20 healthy controls (HCs) to identify the characteristics of the gut microbiome and explore its potential interactions with the host. Decreased microbiota diversity and significant compositional differences were observed in the patients with anemia of CRF. We identified six gut microbiotas significantly changed in the patients with anemia of CRF, particularly *Faecalibacterium prausnitzii*, *Prevotella copri,* and *Escherichia coli*, which were closely correlated with hemoglobin (Hb) levels and estimated glomerular filtration rate (eGFR). These changes were accompanied by functional alterations in distinctive microbial pathways. Further fecal and serum metabolomics revealed fecal 12-KETE-LTB4 in arachidonic acid metabolism, uracil and L-aspartic acid in beta-alanine metabolism, gulonic acid in ascorbate and aldarate metabolism, accompanied by the top 15 differential serum metabolites that were closely correlated with Hb levels. Furthermore, we observed a complex co-occurrence between anemia of CRF-related gut microbiota species and the characterized metabolites. Moreover, a non-invasive model incorporating *Faecalibacterium prausnitzii* and *Prevotella copri*, combined with fecal 12-KETE-LTB4, uracil, L-aspartic acid, and gulonic acid, distinguished the patients with anemia of CRF from HCs (area under the curve: 0.879). Collectively, our results suggest that a disordered gut microbiome associated with functional metabolites may be a non-invasive diagnostic and therapeutic target for anemia of CRF.

**IMPORTANCE:**

Anemia is a prevalent complication in patients with chronic renal failure (CRF), which is associated with a high burden of morbidity and adverse clinical outcomes. Various evidence suggests that gut microbiota dysbiosis may contribute to the pathogenesis of anemia in CRF, although the mechanism is still obscure. This work provides substantial evidence identifying the specific characteristics of the gut microbiomes accompanied by functional alterations in anemia of CRF. We highlight the intricate interactions among the anemia of CRF-related gut microbiome and the functional metabolites, which may regulate toxic accumulation, oxidative stress, and immune-inflammatory responses to induce and exacerbate anemia in patients with CRF. Furthermore, we found that evaluating the gut microbiota and fecal metabolites in combination might be a non-invasive prognostic indicator of CRF-induced anemia. These findings provide important insights into the role of gut microbiota in the mechanism of anemia in CRF.

**CLINICAL TRIALS:**

This study is registered with ClinicalTrials.gov as NCT05543291.

## INTRODUCTION

Chronic renal failure (CRF), characterized by progressive renal damage, affects approximately 700 million people worldwide and represents a severe health risk (1). Anemia is a prevalent complication in patients with CRF and is mainly mediated by increased toxicity, inadequate erythropoietin (EPO) production, inflammation, nutritional deficiencies, and oxidative stress (2). Furthermore, anemia in CRF is associated with a high burden of morbidity and adverse clinical outcomes, which can lead to a reduced lifespan and diminished quality of life for patients (2, 3). Therefore, it is imperative to develop therapeutic interventions and non-invasive tests to identify patients at high risk for anemia of CRF and predisposing factors to slow disease progression.

The gut microbiota maintains a constant dialogue with the host’s essential organ systems, including the kidney (4), bone marrow (5, 6), and cardiovascular system (7, 8). Recently, CRF has been reported to be associated with altered gut microbiome composition and metabolism (4, 9). Certain gut microbiota, such as *Eggerthella lenta*, *Fusobacterium nucleatum,* and *Clostridium sporogenes*, can degrade dietary aromatic amino acids and polyphenols, leading to the production of uremic toxins, including indole-3-sulfate, indole-3-acetic acid, p-cresol sulfate, and hippuric acid (9–11). These gut-derived uremic toxins contribute to the deterioration of renal function and impairment of erythropoiesis (10, 12, 13). Li et al. found that dietary fiber ameliorated renal anemia in patients with end-stage renal disease by restoring levels of *Bifidobacterium adolescentis*, *Lactobacillus*, *Lactobacillaceae,* and short-chain fatty acids (SCFAs) (14). These findings demonstrate the role of gut microbiota and their corresponding metabolites in renal function and anemia, making them novel targets for the precise diagnosis and personalized treatment of anemia of CRF.

The potential mechanisms underlying functional alterations in the gut microbiota and their associated metabolism in anemia of CRF remain unclear. Here, we performed a comprehensive study integrating multidimensional data sets of gut metagenome sequencing and plasma and fecal metabolite profiles in a cohort of patients with anemia of CRF to identify the distinctive characteristics of the gut microbiome and explore its potential interactions with the host.

## RESULTS

### Characteristics of the study population

Thirty patients with anemia of CRF and 20 healthy controls (HCs) were included in the study. Sex and age were matched between the anemia of CRF and HC groups (Table 1). Patients with anemia of CRF exhibited a significantly lower estimated glomerular filtration rate (eGFR) and decreased levels of hemoglobin (Hb) and serum albumin (Alb) compared to those in the HC group (*P* < 0.001; Table 1). No significant differences were found between the two groups with respect to body mass index (BMI), white blood cell counts (WBC), triglycerides (TG), and total cholesterol (TC) (*P* > 0.05, Table 1).

**TABLE 1:** Clinical characteristics of study populations^*a*^

Characteristic	HC (*n* = 20)	CRF-anemia (*n* = 30)	*P* value
Male, *n* (%)	9 (45.0)	14 (46.7)	0.908
Mean age (years) ± SD	62.65 ± 8.33	61.13 ± 10.33	0.853
CRF causes, *n* (%)			
DN		11 (36.67)	
CGN		13 (43.33)	
Others		6 (20.0)	
Mean BMI (kg/m^2^) ± SD	23.47 ± 2.37	23.66 ± 3.44	0.736
eGFR (mL/min/1.73 m^2^), median (IQR)	104.48 (97.15–110.81)	9.98 (5.70–16.56)	<0.001
Mean WBC (× 10^12^/L) ± SD	5.66 ± 1.38	5.40 ± 1.57	0.501
Hb (g/L), median (IQR)	143.00 (135.25–155.75)	90.50 (82.00–98.50)	<0.001
Alb (g/L), median (IQR)	48.40 (46.98–51.30)	36.95 (33.60–40.78)	<0.001
TC (mg/dL), median (IQR)	4.55 (4.34–5.26)	3.96 (3.50–5.10)	0.080
TG (mg/dL), median (IQR)	1.54 (1.08–1.97)	1.64 (1.29–2.52)	0.513

^*a*^ CRF, chronic renal failure; HC, healthy control; SD, standard deviation; DN, diabetic nephropathy; CGN, chronic glomerulonephritis; BMI, body mass index; IQR, interquartile range; eGFR, estimated glomerular filtration rate; WBC, white blood cell counts; Hb, hemoglobin; Alb, albumin; TG, triglycerides; TC, total cholesterol.

### Disturbed fecal microbiota in patients with anemia of CRF

**Fig 1:**
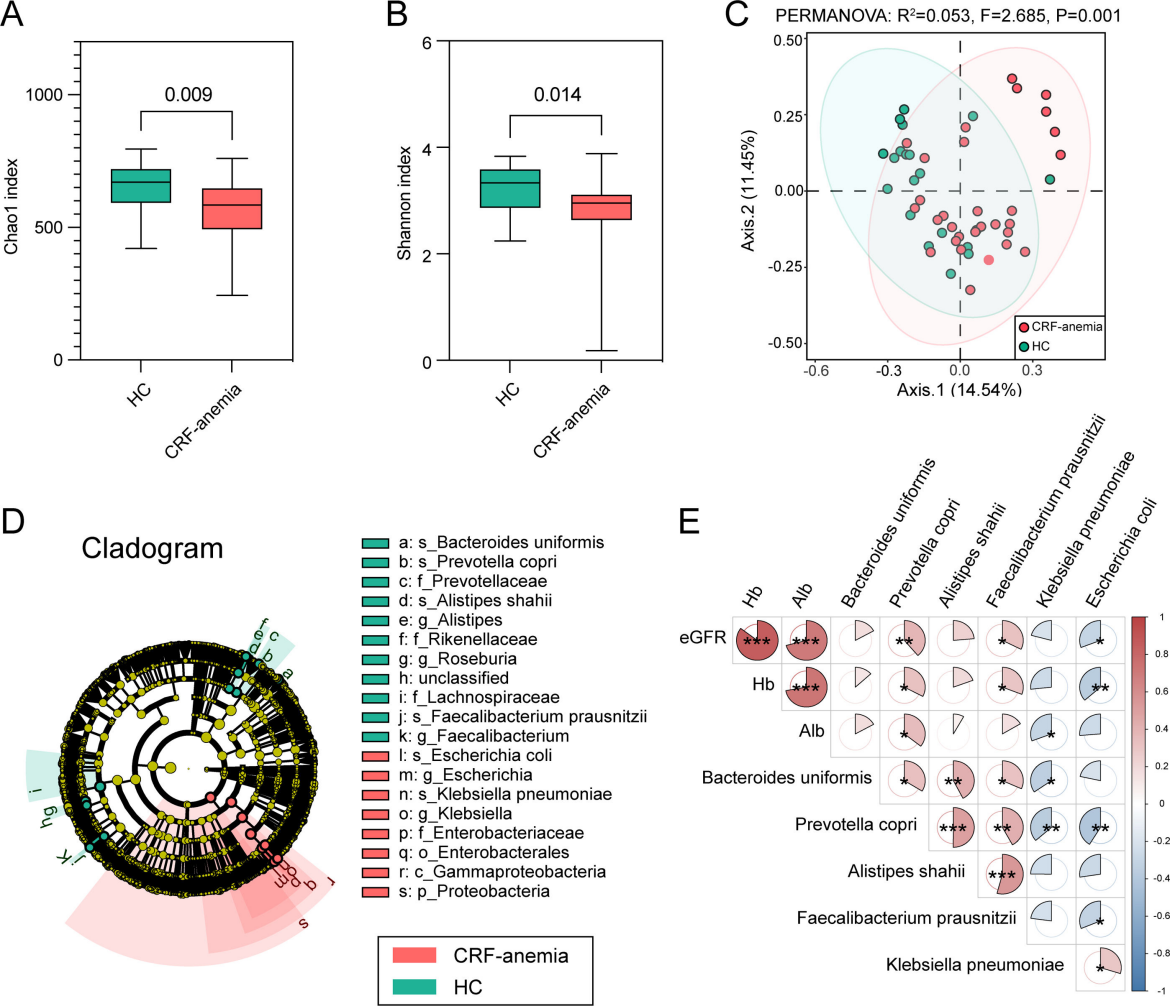
Alterations of gut microbiota in patients with anemia of CRF and its correlation with clinical indices. Comparisons of alpha diversity using the (**A**) Chao1 index and (**B**) Shannon index between patients with anemia of CRF and HC groups. (**C**) PCoA utilizing the Bray-Curtis dissimilarity metric revealed substantial variation in the beta diversity between patients with anemia of CRF and HCs. (**D**) A LEfSe analysis highlighted a differential abundance of bacterial taxa between patients with anemia of CRF and HCs (LDA score > 4.0, *P* < 0.050). (**E**) Correlation heatmap depicting the relationship between specific microbial species and clinical parameters (*P < 0.050, **P < 0.010, ***P < 0.001). Negative correlations are represented by blue shading, while positive correlations are indicated by red shading, with the areas of the sector corresponding to the magnitude of the correlation coefficient (*r*). CRF, chronic renal failure; HC, healthy control; eGFR, estimated glomerular filtration rate; Hb, hemoglobin; Alb, albumin.

We performed a fecal metagenomic analysis in patients with anemia of CRF and HCs to explore the link between the gut microbiome and CRF-induced anemia. A significant reduction in the alpha diversity assessed by Chao1 (584.0 vs 670.0, *P* = 0.009; Fig. 1A) and Shannon indices (2.955 vs 3.330, *P* = 0.014; Fig. 1B) was detected in the fecal microbiota of patients with anemia of CRF. The beta diversity analysis revealed a distinct composition of microbial communities between the two groups based on the principal coordinate analysis (PCoA) (*P* = 0.001 with permutational analysis of variance [PERMANOVA] of Bray-Curtis distances; Fig. 1C).

Based on the linear discriminant effect size (LEfSe), considerable differences in the gut microbial profiles between the anemia of CRF and HC groups were observed (Fig. 1D). The abundance of the phylum *Proteobacteria* was enriched in patients with anemia of CRF, particularly those belonging to the *Gammaproteobacteria* class and *Enterobacteriaceae* family, including *Escherichia coli* and *Klebsiella pneumoniae* (linear discriminant analysis [LDA] score > 4.0, *P* < 0.050; Fig. 1D). Meanwhile, *Bacteroides uniformis; Prevotella copri* and its family *Prevotellaceae; Alistipes shahii* and its family *Rikenellaceae; Faecalibacterium prausnitzii* and its genus *Faecalibacterium*; and *Roseburia* and its family *Lachnospiraceae* were more abundant in HCs (LDA score > 4.0, *P* < 0.050; Fig. 1D). Notably, the gut microbiota was closely associated with Hb levels and other clinical indicators (Fig. 1E). In particular, *P. copri* and *F. prausnitzii* were positively correlated with Hb levels (*P. copri*, *r* = 0.349, *P* = 0.013; *F. prausnitzii*, *r* = 0.329, *P* = 0.020) and eGFR (*P. copri*, *r* = 0.400, *P* = 0.004; *F. prausnitzii*, *r* = 0.335, *P* = 0.018), whereas *E. coli* showed an opposite relationship (Hb, *r* = –0.334, *P* = 0.008; eGFR, *r* = –0.272, *P* = 0.049). Meanwhile, serum Alb levels were positively correlated with *P. copri* (*r* = 0.373, *P* = 0.018) but negatively correlated with *K. pneumoniae* (*r* = –0.285, *P* = 0.045). In summary, anemia of CRF showed significant alterations in six gut microbiotas, particularly *P. copri*, *F. prausnitzii*, and *E. coli*, and these changes were consistent with eGFR.

**Fig 2:**
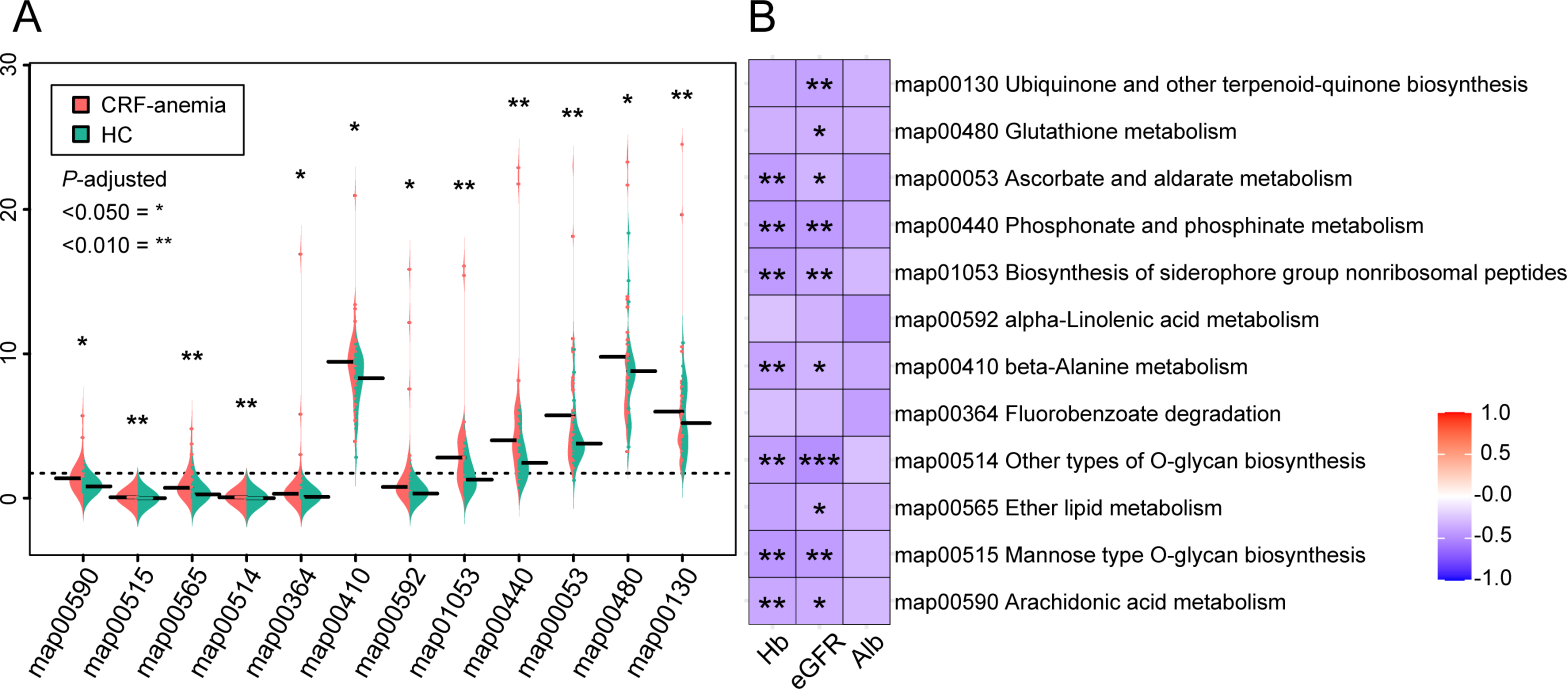
Changes in gut microbiota metabolic function and its correlation with Hb levels in patients with anemia of CRF. (**A**) Significant differences in metabolic Kyoto Encyclopedia of Genes and Genomes (KEGG) pathways between patients with anemia of CRF and healthy controls (*P < 0.05, **P < 0.010; *P* value was determined by Wilcoxon rank-sum test with FDR correction). (**B**) Heatmaps of differentially expressed metabolic pathways and clinical indices, including hemoglobin, estimated glomerular filtration rate, and serum albumin (*P < 0.050, **P < 0.010, ***P < 0.001). Negative correlations are indicated in purple, while positive correlations are shown in red. CRF, chronic renal failure; HC, healthy control; eGFR, estimated glomerular filtration rate; Hb, hemoglobin; Alb, albumin.

We also investigated the roles of microorganisms and their associated metabolic pathways in anemia of CRF. Twelve distinct metabolic pathways were identified in the anemia of CRF group that were mostly involved in uremic toxin production, inflammation, and oxidative stress (*P* < 0.05; Fig. 2A). Of these, the seven essential metabolic pathways involved, ascorbate and aldarate metabolism (map00053), phosphonate and phosphinate metabolism (map 00440), biosynthesis of siderophore group nonribosomal peptides (map01053), beta-alanine metabolism (map00410), other types of O-glycan biosynthesis (map00514), mannose type O-glycan biosynthesis (map00515), and arachidonic acid metabolism (map00590), were inversely correlated with Hb levels and eGFR (*P* < 0.05; Fig. 2B). In addition, ubiquinone and other terpenoid-quinone biosynthesis (map00130), glutathione metabolism (map00480), and ether lipid metabolism (map00565) were linked to eGFR only (*P* < 0.05; Fig. 2B). These findings suggest that the dysregulation of the seven essential microbial metabolic processes contributes to anemia of CRF.

### Fecal and plasma metabolomic alterations in patients with anemia of CRF

**Fig 3:**
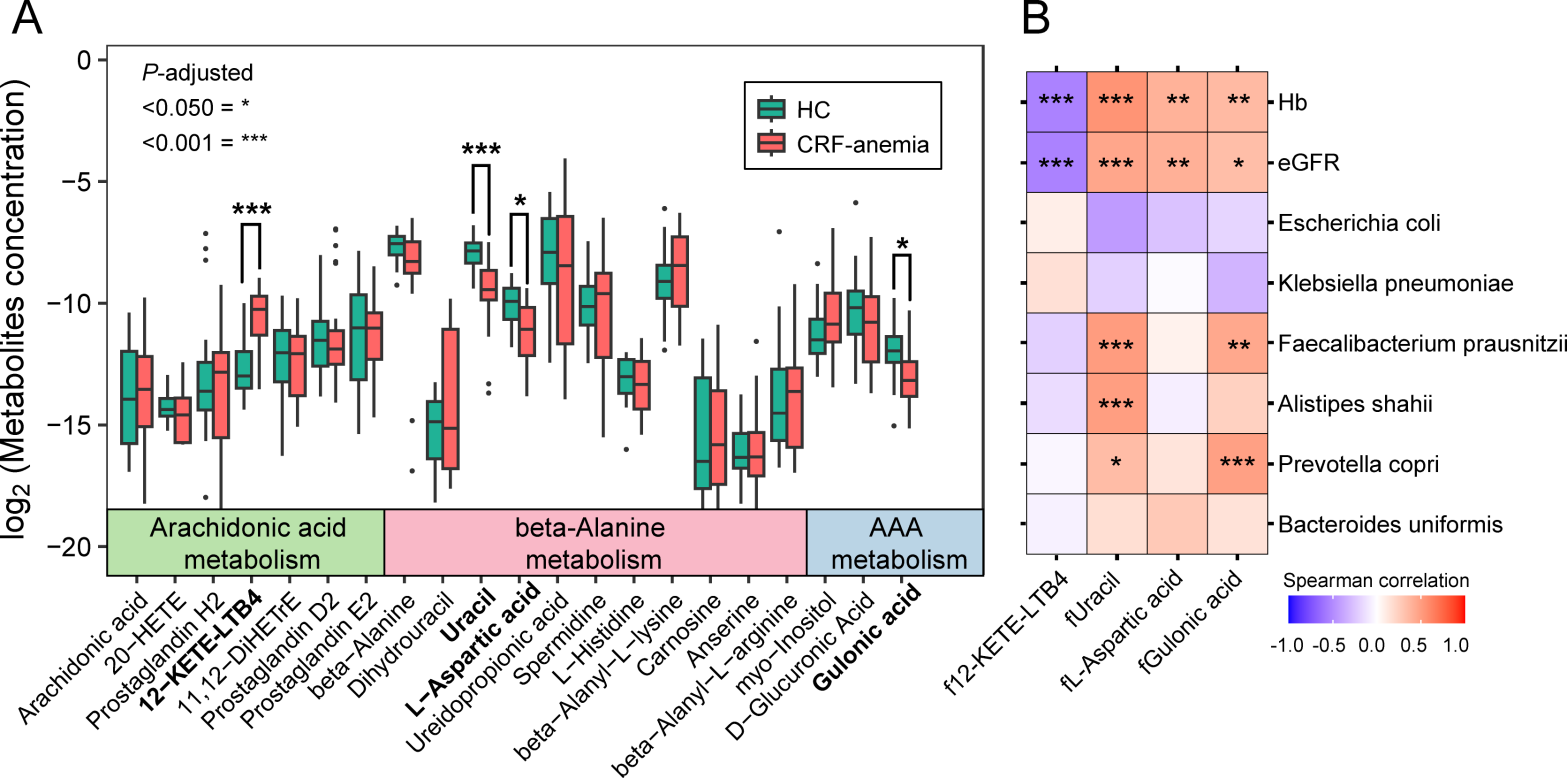
Changes in gut microbiota-linked fecal metabolites in patients with anemia of CRF. (**A**) Comparative analysis of fecal metabolites between anemia of CRF and HC groups (*P < 0.05, ***P < 0.001; *P* value was determined by Wilcoxon rank-sum test with FDR correction). (**B**) Heatmaps display the correlation between clinical indices, gut microbiota, and fecal metabolites (*P < 0.050, **P < 0.010, ***P < 0.001). Negative correlations are denoted by purple shading, and positive correlations are represented in red. CRF, chronic renal failure; HC, healthy control; AAA metabolism, ascorbate and aldarate metabolism; 12-KETE-LTB4, 12-keto-tetrahydro-leukotriene B4; eGFR, estimated glomerular filtration rate; Hb, hemoglobin.

To determine gut microbiota-associated metabolic disturbances in patients with anemia of CRF, we further analyzed fecal metabolites related to the seven essential metabolic pathways using untargeted liquid chromatography (LC)–mass spectrometry (MS). Based on untargeted fecal metabolomics, 21 of the 975 annotated fecal metabolites involved in arachidonic acid, beta-alanine, ascorbate, and aldarate metabolism were identified (*P* < 0.05; Table S1). Among these, 12-keto-tetrahydro-leukotriene B4 (12-KETE-LTB4), involved in arachidonic acid metabolism, was markedly elevated in anemia of CRF; however, the opposite was found for uracil, L-aspartic acid, and gulonic acid, which are involved in ascorbate, aldarate, and beta-alanine metabolism (*P* < 0.05; Fig. 3A). We then assessed the relationships between these four fecal metabolites, eGFR, Hb, and anemia of CRF-related gut microbiota. Notably, these fecal metabolites significantly correlated with Hb levels in patients with anemia of CRF. For example, fecal 12-KETE-LTB4 levels were inversely correlated with Hb and eGFR (Hb, *r* = –0.540, *P* < 0.001; eGFR, *r* = –0.540, *P* < 0.001). In contrast, metabolites enriched in the HC group, including uracil (Hb, *r* = 0.549, *P* < 0.001; eGFR, *r* = 0.460, *P* < 0.001), L-aspartic acid (Hb, *r* = 0.464, *P* = 0.005; eGFR, *r* = 0.475, *P* = 0.004), and gulonic acid (Hb, *r* = 0.363, *P* = 0.009; eGFR, *r* = 0.341, *P* = 0.015), were positively correlated with Hb levels and eGFR (Fig. 3B). Furthermore, Spearman’s correlation analysis indicated that fecal uracil and gulonic acid were strongly positive with anemia of CRF-related gut microbiota species, such as *P. copri* (uracil, *r* = 0.348, *P* = 0.013; gulonic acid, *r* = 0.486, *P* < 0.001), *A. shahii* (uracil, *r* = 0.504, *P* < 0.001), and *F. prausnitzii* (uracil, *r* = 0.510, *P* < 0.001; gulonic acid, *r* = 0.450, *P* = 0.001; Fig. 3B).

**Fig 4:**
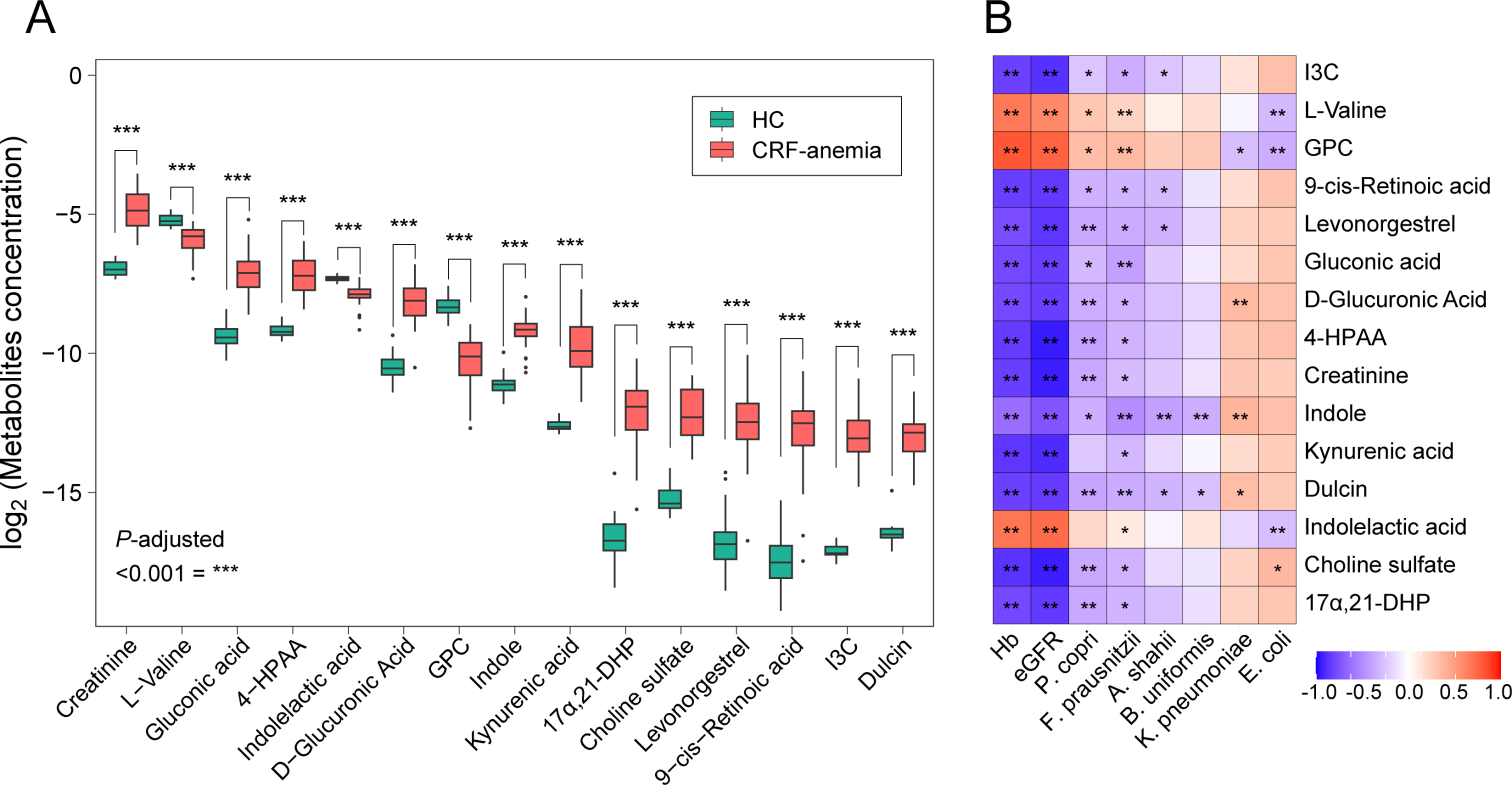
Changes in serum metabolites in patients with anemia of CRF. (**A**) Top 15 representative differential serum metabolites in patients with anemia of CRF compared to the healthy control (***P < 0.001; *P* value was determined by Wilcoxon rank-sum test with FDR correction). (**B**) Heatmaps display the correlation between clinical indices, gut microbiota, and serum metabolites (*P < 0.050, **P < 0.010, ***P < 0.001). Negative correlations are indicated in blue, while positive correlations are shown in red. CRF, chronic renal failure; HC, healthy control; eGFR, estimated glomerular filtration rate; Hb, hemoglobin; 17α,21-DHP, 17α,21-dihydroxypregnenolone; 4-HPAA, 4-hydroxyphenylacetaldehyde; GPC, glycerophosphocholine; I3C, indole-3-carboxylic acid.

Next, we analyzed the serum metabolome to delineate specific alterations in the host. Compared to those in the HC group, patients with anemia of CRF exhibited 167 elevated serum metabolites and 258 reduced metabolites (*P* < 0.050, Table S2). Here, we highlighted the top 15 serum metabolites identified by univariate statistical analysis. Among these metabolites, the levels of creatinine, gluconic acid, 4-hydroxyphenylacetaldehyde, D-glucuronic acid, indole, kynurenic acid, 17alpha,21-dihydroxypregnenolone, choline sulfate, levonorgestrel, 9-cis-retinoic acid, indole-3-carboxylic acid, and dulcin were remarkably higher in the anemia of CRF group than in the HC group, whereas the serum L-valine, indolelactic acid, and glycerophosphocholine levels showed the opposite trend (*P* < 0.001; Fig. 4A). Moreover, these serum metabolites showed a robust correlation with anemia of CRF-related gut microbiota species, particularly *P. copri* and *F. prausnitzii*, along with Hb levels and eGFR (*P* < 0.05; Fig. 4B).

### Association between gut microbiota and fecal and plasma metabolites

**Fig 5:**
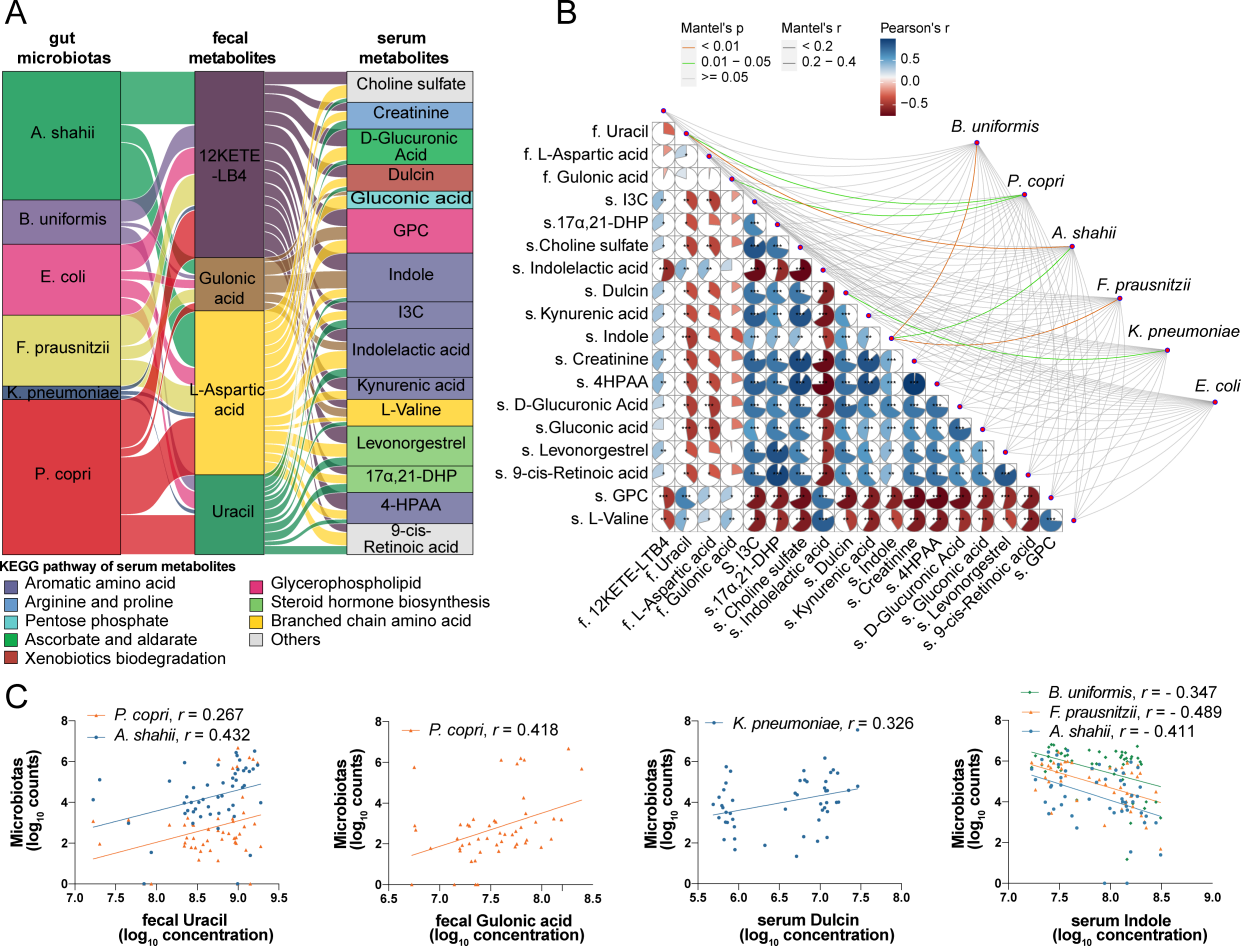
Complex interactions between anemia of CRF-related gut microbiota and functional metabolites in the feces and serum. (**A**) Sankey diagram focused on gut dysbiosis and the relationship among fecal and serum metabolites. (**B**) Mantel test results examining gut microbial species and characterized functional metabolites (*P < 0.050, **P < 0.010, ***P < 0.001). Metabolites prefixed with a lowercase “f” represent fecal metabolites, whereas those prefixed with a lowercase “s” denote serum metabolites. Negative correlations are indicated in blue, and positive correlations are indicated in red. (**C**) Compilation of scatter plots depicting correlations for features with statistically significant associations, as determined by the Mantel test. CRF, chronic renal failure; *B. uniformis*, *Bacteroides uniformis; P. copri*, *Prevotella copri*; *A. shahii*, *Alistipes shahii; F. prausnitzii*, *Faecalibacterium prausnitzii; K. pneumoniae*, *Klebsiella pneumoniae; E. coli*, *Escherichia coli*; 12-KETE-LTB4, 12-keto-tetrahydro-leukotriene B4; eGFR, estimated glomerular filtration rate; Hb, hemoglobin; 17α,21-DHP, 17α,21-dihydroxypregnenolone; 4-HPAA, 4-hydroxyphenylacetaldehyde; GPC, glycerophosphocholine; I3C, indole-3-carboxylic acid.

Based on the metagenomic and metabolomic data, we performed a co-occurrence analysis to investigate the putative interactions between the gut microbiota and functional metabolites in anemia of CRF. The Sankey diagram revealed a complex co-occurrence network involving six aberrant gut microbiota, four altered fecal metabolites, and 15 disturbed serum metabolites (*P* < 0.05; Fig. 5A). Notably, a strong correlation between the anemia of CRF-related gut microbiota and the characterized metabolites was noted (*P* < 0.05; Fig. 5B). For example, *P. copri* was positively associated with gulonic acid (*r* = 0.418, *P* = 0.033; Fig. 5C) and uracil (*r* = 0.267, *P* = 0.046; Fig. 5C). Similarly, *A. shahii* was positively correlated with fecal uracil (*r* = 0.432, *P* = 0.002; Fig. 5C) yet was negatively correlated with serum indole (*r* = –0.411, *P* = 0.037; Fig. 5C). In contrast, both *F. prausnitzii* (*r* = –0.489, *P* < 0.001; Fig. 5C) and *B. uniformis* (*r* = –0.347, *P* = 0.007; Fig. 5C) were inversely associated with serum indole. Moreover, *K. pneumoniae* was positively correlated with serum dulcin levels (*r* = 0.326, *P* = 0.021; Fig. 5C). These results highlight the role of gut dysbiosis in the disruption of host metabolism, which may exacerbate anemia of CRF.

### Identification of non-invasive classifier for anemia of CRF

**Fig 6:**
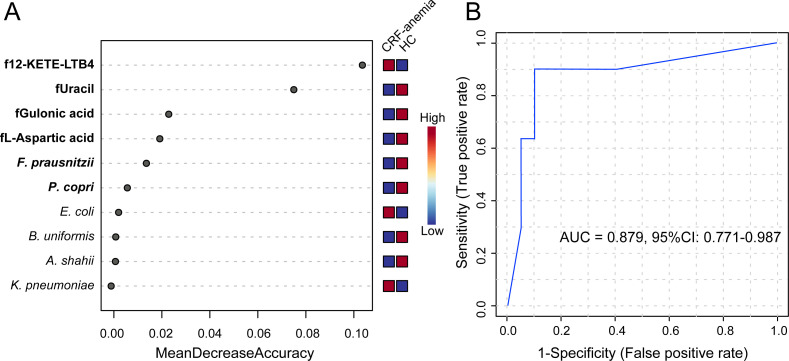
The potential for non-invasive diagnosis of anemia of CRF based on fecal microbiota and metabolites. (**A**) Features are ranked based on their contributions to classification accuracy (mean decrease accuracy) using the Random Forest algorithm. (**B**) The receiver operating characteristic curve for anemia of CRF, utilizing the combination of two microbial species (*Prevotella copri* and *Faecalibacterium prausnitzii*) and four fecal metabolites (12-keto-tetrahydro-leukotriene B4, uracil, L-aspartic acid, and gulonic acid). CRF, chronic renal failure; HC, healthy control; *B. uniformis*, *Bacteroides uniformis*; *P. copri*, *Prevotella copri*; *A. shahii*, *Alistipes shahii; F. prausnitzii*, *Faecalibacterium prausnitzii; K. pneumoniae*, *Klebsiella pneumoniae; E. coli*, *Escherichia coli*; 12-KETE-LTB4, 12-keto-tetrahydro-leukotriene B4; AUC, area under the curve; 95% CI, 95% confidence interval.

To assess the utility of gut microbiota and fecal metabolite alterations as a non-invasive diagnostic tool for anemia of CRF, we first performed a univariate linear regression analysis to evaluate their effectiveness in identifying this condition. *P. copri* and *F. prausnitzii* were identified as the most relevant microbial species associated with the levels of Hb and eGFR (*P* < 0.05; Table 2). We also selected four fecal metabolites closely correlated with Hb levels: 12-KETE-LTB4, uracil, L-aspartic acid, and gulonic acid (*P* < 0.05; Table 2). Notably, 12-KETE-LTB4, uracil, L-aspartic acid, and gulonic acid combined with *P. copri* and *F. prausnitzii* provided the most significant discriminatory power to distinguish anemia of CRF from HCs using the Random Forest model (mean decrease accuracy > 0.005, Fig. 6A). Following a 10-fold cross-validation, the combination of these four metabolites and two microbial taxa showed a significant ability to diagnose anemia of CRF, with an area under the curve of 0.879 (95% confidence interval [CI]: 0.771–0.987, Fig. 6B).

**TABLE 2:** Univariate linear regression analysis of characteristic gut microbiotas and fecal metabolites in CRF patients with Hb and eGFR^*a*^

Variable	Hb		eGFR	
	β (95% CI)	*P* value	β (95% CI)	*P* value
*B. uniformis*	3.768 (–2.599, 10.134)	0.240	9.037 (–0.255, 18.328)	0.056
*P. copri*	8.203 (3.195, 13.211)	0.002	12.766 (5.353, 20.180)	0.001
*A. shahii*	5.235 (–0.756, 11.226)	0.085	10.851 (2.166, 19.535)	0.015
*F. prausnitzii*	8.509 (0.302, 16.717)	0.042	18.409 (6.766, 30.052)	0.003
*K. pneumoniae*	–6.386 (–13.186, 0.4114)	0.065	–10.118 (–20.235, 0.102)	0.139
*E. coli*	–6.551 (–13.588, 0.487)	0.067	–7.758 (–18.419, 2.902)	0.150
12-KETE-LTB4	–41.404 (–58.290, –24.518)	<0.001	–66.518 (–90.743, –42.293)	<0.001
Uracil	36.715 (20.654, 52.775)	<0.001	54.101 (29.949, 78.252)	<0.001
L-Aspartic acid	38.883 (18.383, 59.382)	0.009	61.080 (30.931, 91.230)	0.002
Gulonic acid	35.008 (14.462, 55.554)	0.001	51.446 (20.613, 82.278)	0.002

^*a*^ CRF, chronic renal failure; eGFR, estimated glomerular filtration rate; Hb, hemoglobin; *B. uniformis*, *Bacteroides uniformis*; *P. copri*, *Prevotella copri*; *A. shahii*, *Alistipes shahii; F. prausnitzii*, *Faecalibacterium prausnitzii; K. pneumoniae*, *Klebsiella pneumoniae; E. coli*, *Escherichia coli*; 12-KETE-LTB4, 12-keto-tetrahydro-leukotriene B4; 95% CI, 95% confidence interval.

## DISCUSSION

Accumulating evidence suggests that alterations in the gut microbiome and metabolomics are central to the development of CRF (4, 9, 10, 15). Gut dysbiosis and its associated products, such as phenol, p-cresol, and trimethylamine, contribute to the uremic load and exacerbate CRF progression (4, 9, 13, 16). However, the functional potential of the gut microbiome in patients with anemia of CRF, a prevalent complication, and its complex interplay with host metabolism remain underestimated. In the present study, we profiled the altered gut microbiota and metabolome, along with their potential interactions with disordered host metabolism, in a cohort of patients with anemia of CRF. We identified gut microbiota dysbiosis and disruptions of the fecal and serum metabolomes that may regulate toxic accumulation, oxidative stress, and immune–inflammatory responses to induce and exacerbate anemia in patients with CRF. Additionally, we found that evaluating the gut microbiota and fecal metabolites in combination might be a non-invasive prognostic indicator of anemia of CRF.

Gut microbiota dysbiosis occurs in patients with CRF and may contribute to anemia and renal dysfunction (6, 17). The abundance of the potentially pathogenic *Proteobacteria* and the decrease in *Roseburia* spp. and *F. prausnitzii* are associated with CRF progression (15, 18); however, the mechanisms underlying the association between the gut microbiota and anemia of CRF remain unclear. In this study, we identified specific taxonomic alterations in patients with anemia of CRF, noting that *F. prausnitzii* and *P. copri* were positively correlated with both Hb levels and eGFR, whereas *E. coli* showed the opposite trend. Importantly, *F. prausnitzii* is a beneficial bacterium that produces SCFAs, which, evidence suggests, are associated with inhibiting inflammation, attenuating kidney injury, and stimulating the differentiation of erythroid progenitor cells (15). Notably, butyrate derivatives have been effectively used to treat anemia (19, 20). Furthermore, in the present study, we also observed a decrease in the abundance of *P. copri* and its associated *Prevotellaceae* family members in patients with anemia of CRF. Previous research has indicated that *P. copri* can alleviate liver fibrosis and regulate immunity in patients with severe aplastic anemia (21, 22), and its homologous species, *Prevotella 9*, is positively correlated with EPO levels (6). Therefore, anemia of CRF may be caused by decreased SCFAs produced by *F. prausnitzii* and decreased EPO production mediated by *P. copr*i through immune mediation, thereby inhibiting erythroid progenitor cell growth. Consistent with previous studies, we found a negative correlation between *E. coli* and the severity of anemia in patients with CRF. *E. coli*, a member of the *Proteobacteria* phylum, specifically the *Gammaproteobacteria* class and *Enterobacteriaceae* family, is a predominantly facultative anaerobic and opportunistic pathogen. This organism aggravates calcium oxalate stone formation via PPK1/flagellin-mediated renal oxidative injury and inflammation (23). *E. coli* also exhibits enhanced activity in pathways related to energy metabolism, particularly the glycolytic pathway (24). As the host initiates immune signaling to combat infection, intestinal epithelial cells respond to aerobic glycolysis by rapidly mobilizing bioenergetic molecules, leading to epithelial oxygenation, a spike in mucosal-associated commensal *Enterobacteriaceae,* and a concurrent decline in obligate anaerobes (25). Ultimately, despite the limited research on the role of *E. coli* in anemia of CRF, it has been proposed that the bacterium could potentially foster anemia by modulating inflammation and intestinal epithelial function.

The Kyoto Encyclopedia of Genes and Genomes (KEGG) pathway analysis based on metagenomics revealed that, despite ubiquinone and other terpenoid-quinone biosynthesis, glutathione metabolism, and ether lipid metabolism being associated with eGFR alone, anemia was more closely associated with seven distinctive metabolic pathways. Among these pathways, arachidonic acid, ascorbate, aldarate, and beta-alanine metabolism were the most important. Notably, several studies have demonstrated the roles of arachidonic acid, ascorbate, and aldarate metabolism in hematopoietic cell proliferation and differentiation (26, 27); however, their specific effects on the development of renal anemia are not well understood. In our analyses, fecal metabolomic analyses identified four distinct metabolites, 12-KETE-LTB4, uracil, L-aspartic acid, and gulonic acid, from three essential metabolic pathways in anemia of CRF. Specifically, we found that 12-KETE-LTB4, which is involved in arachidonic acid metabolism, was inversely correlated with Hb levels. Moreover, 12-KETE-LTB4 is a known signaling molecule involved in lipopolysaccharide-induced immune stress, neuroactive ligand–receptor interactions, and the peroxisome proliferator-activated receptor (PPAR) signaling pathway (28). Furthermore, it is crucial to highlight that the downregulation of cecal 12-KETE-LTB4 in broilers alleviates intestinal barrier damage and intestinal inflammation caused by immune stress (28). These findings highlight the complex interplay between specific metabolic pathways and anemia in CRF, highlighting the need for further research to clarify the mechanisms linking gut-derived metabolites to hematopoietic health.

Our findings indicated that beta-alanine metabolites were depleted in the fecal matter (e.g., L-aspartic acid and uracil) of patients with anemia of CRF. Specifically, L-aspartic acid, a precursor in the synthesis of asparagine, plays a crucial role in regulating immunity and inflammation (29). In addition, uracil generated in the mitochondria has been identified as a pharmacodynamic biomarker for evaluating the therapeutic effect of traditional Chinese medicines on anemia (30). Gulonic acid in ascorbate and aldarate metabolism is a precursor of ascorbic acid, which is considered a non-essential vitamin that contributes to antioxidation and immune function (31). These findings suggest that changes in the gut microbiota in anemia of CRF and metabolic dysregulation may be linked to imbalances in oxidative stress, inflammation, and intestinal mucosal barrier integrity. However, the precise regulatory mechanisms underlying these changes in the hematopoietic system require further investigation.

In this study, we identified that a range of disrupted plasma metabolites correlated with Hb levels in patients with anemia of CRF, including those involved in aromatic amino acid metabolism, arginine and proline metabolism, branched-chain amino acid metabolism, ascorbate and aldarate metabolism, xenobiotic biodegradation and metabolism, the pentose phosphate pathway, steroid hormone biosynthesis, and glycerophospholipid metabolism (Fig. 5A). Remarkably, we highlighted the complex interactions between six anemia of CRF-related microbial species, four characteristic fecal metabolites, and 15 blood metabolites. The levels of fecal uracil and gluonic acid were positively associated with the anemia of CRF-related microbial species *P. copri* and *A. shahii*, which may be attributed to the compromised antioxidant capacity and excessive inflammatory and immune reactions in the gut of patients with anemia of CRF (20–22). Furthermore, indole, which is catabolized from tryptophan, was increased in the serum of patients with anemia of CRF and showed a negative relationship with *B. uniformis, F. prausnitzii,* and *A. shahii*. Notably, *B. uniformis* serves as a fiber-degrading microbiota capable of amplifying butyrate production from dietary fibers (32). Together, these results suggest that these three microbial species promote the production of SCFAs through a synergistic effect and regulate tryptophan metabolism through cross-feeding, thereby inhibiting the production of harmful indole. Additionally, our findings also identified that dulcin, an aromatic compound also known as p-ethoxyphenylurea with toxic side effects (33), is positively associated with the opportunistic pathogen *K. pneumoniae* and negatively correlated with both Hb and eGFR levels, indicating its potentially toxic effects in anemia. Overall, growing evidence suggests that aromatic amino acids contribute to the production of major uremic toxins, which may advance renal fibrosis and erythropoiesis in CRF (9, 13). Thus, we speculate that these microbial communities may affect hematopoietic cell function through these metabolites, thereby influencing anemia caused by renal dysfunction; however, further investigation is required to identify the potential mechanisms.

In the present study, we revealed that individuals with CRF are likely to have anemia or prevent its occurrence due to the influence of their intestinal microbiota and metabolites. Here, we established a non-invasive model to assess the risk of anemia of CRF using linear regression and Random Forest analyses. The integration of gut microbial and metabolomic data into a diagnostic framework indicated that the presence of *P. copri*, *F. prausnitzii*, 12-KETE-LTB4, uracil, L-aspartic acid, and gulonic acid plays a key role in the pathogenesis of anemia of CRF and provides a promising non-invasive approach for its early detection and intervention in the clinic.

It is important to acknowledge that our study has some limitations. First, the study did not include patients with CRF without anemia or analyze patients with different anemia severity. This is mainly due to the scarcity of such patients in the renal failure stage, especially those with an eGFR < 20 mL/min/1.73 m^2^. Instead, we conducted a correlation analysis of the degree of anemia, intestinal bacteria, and metabolites. Therefore, future studies are needed on the dynamic evolution of meta-transcriptomic profiles in anemia of CRF and to explore the underlying potential mechanisms. Second, owing to the small sample size of the study, the robustness and generalizability of this model in identifying anemia of CRF require further exploration in broader and more diverse patient populations.

### Conclusion

Our study provides insights into the role of altered fecal microbial and metabolic profiles in anemia of CRF. These findings have two important implications. First, anemia of CRF was associated with distinct changes in the gut microbiota, particularly in the abundances of *P. copri*, *F. prausnitzii*, and *E. coli,* accompanied by specific metabolic disturbances in both the gut and host, and these changes were consistent with both Hb levels and eGFR. Second, *P. copri* and *F. prausnitzii* combined with fecal 12-KETE-LTB4, uracil, L-aspartic acid, and gulonic acid provided a potential prognostic indicator for the non-invasive assessment of the risk of anemia of CRF; however, this warrants further validation through a comprehensive analysis within an expanded study population. Targeted interventions that modulate the gut microbiome and metabolic profile may represent novel strategies for the prevention and treatment of anemia of CRF.

## MATERIALS AND METHODS

### Study population and sample collection

Patients with anemia of CRF and HCs were recruited from June 2021 to December 2021 in the Nephrology Unit of Shanghai Tenth People’s Hospital affiliated with Tongji University, Shanghai, China. The inclusion criteria for the anemia of CRF group were as follows: (i) patients 18–80 years old, (ii) patients with a diagnosis of CRF (EPI eGFR < 20 mL/min/1.73 m^2^) by a nephrology specialist (34), and (iii) patients with an Hb < 130 g/L in males and <120 g/L in females (35). Adults with normal renal function matched by age and gender were included in the HC group. Subjects with the following conditions were excluded: (i) administration of any antibiotic, probiotic, glucocorticoids, immunosuppressive agents, or antineoplasm treatments 1 mo before fecal sample collection; (ii) active gastrointestinal diseases, such as active inflammatory bowel disease, irritable bowel syndrome, and diarrhea; (iii) anemia unrelated to CRF (e.g., Mediterranean anemia, aplastic anemia, gastrointestinal bleeding, or hemolytic anemia); (iv) active viral hepatitis, tuberculosis, and other infectious diseases; (v) solid tumors or hematologic tumors; (vi) history of hemorrhage within the past 3 mo; (vii) pregnancy; and (viii) receipt of renal replacement therapy.

Clinical information, including sex, age, cause of CRF, BMI, eGFR, WBC, Hb, Alb, TG, and TC, was collected from the medical records. Fecal and serum samples from the same participants were collected on the same day. Serum samples were centrifuged at 3,000 rpm at 4°C for 10 min. The supernatants and fecal samples were stored at –80°C before further processing.

### DNA extraction and metagenomic sequencing

DNA from stool samples was isolated using the hexadecyl trimethyl ammonium bromide (CTAB) method with CTAB extraction solution (N0211, Nobleryder, China). DNA degradation degree, potential contamination, and concentration were measured using an Agilent Fragment Analyzer 5400 (Agilent, USA). Sequencing libraries were constructed using the NEBNext Ultra DNA Library Prep Kit (E7370L, NEB, USA), following the manufacturer’s recommendations, and index codes were added to each sample. The prepared libraries underwent paired-end sequencing (150 bp) on the Illumina NovaSeq 6000 platform. Raw sequencing data were obtained through metagenomic sequencing. To ensure the reliability of data, the raw sequencing data were preprocessed using Kneaddata software for quality control (QC) based on the Trimmomatic tool and host DNA removal based on the Bowtie2 tool. Finally, the clean reads were exported for microbiome data analysis described below.

### Fecal and plasma sample preparation for metabolomics

To process the fecal samples, the following steps were taken: after thawing at 4°C, about 100 mg of each sample was weighed and mixed with 600 µL of methanol containing 2-chlorophenylalanine at a concentration of 4 ppm for 30 s using a vortex mixer set at –20℃. This was followed by sonication for 10 min, after which the sample was ground with 100 mg of glass beads for 90 s at a frequency of 60 Hz. Subsequently, the mixture was centrifuged at 12,000 rpm for 10 min at 4°C, and approximately 300 µL of the supernatant was carefully collected after filtering through a 0.22 µm membrane. For the serum samples, 100 µL of each sample was combined with 400 µL of a 50% ice-cold methanol solution. Following centrifugation at 12,000 rpm for 10 min at 4°C, the supernatant was carefully transferred to a new 2 mL centrifuge tube and concentrated to dryness under vacuum conditions. Subsequently, the samples were redissolved in 150 µL of an 80% methanol solution containing 2-chlorophenylalanine at a concentration of 4 ppm. Afterward, the supernatant was passed through a 0.22 µm filter in preparation for LC–MS analysis. The QC samples were prepared by mixing 20 µL of supernatant from each sample. All formal samples and QC samples were then utilized for non-targeted metabolomics analysis via LC–MS.

A Vanquish ultra-high-performance liquid chromatography system (Thermo Fisher Scientific) was used for the LC–MS analysis. For positive polarity, the mobile phases comprised eluent A2 (0.1% formic acid in water) and eluent B2 (0.1% formic acid in acetonitrile); for negative polarity, they consisted of eluent A3 (5 nM ammonium fluoride in water) and eluent B3 (acetonitrile). A linear gradient of eluent B2/B3 (volume ratio) was applied as follows: 0–1 min, 2%; 1–9 min, 2%–50%; 9–12 min, 50%–98%; 12–13.5 min, 98%; 13.5–14 min, 98%–2%; and 14–20 min, 2%. The injection volume was 2 µL at a flow rate of 0.25 mL/min. Electrospray ionization tandem mass spectrometry was performed on a Thermo Fisher Q Exactive mass spectrometer using a spray voltage of 3.5 kV for positive mode and −2.5 kV for negative mode. The source conditions included a sheath gas flow rate of 30 arbitrary units, auxiliary gas flow rate of 25 arbitrary units, capillary temperature of 325℃, full scan range from 81 to 1,000 m/z, mass resolution of 7,000, and normalized collision energy of 30 eV. Data-dependent acquisition MS/MS experiments were conducted using higher-energy collisional dissociation (HCD) scans. Dynamic exclusion was employed to filter out superfluous information from the MS/MS spectra (36). ProteoWizard software was used to convert raw data to mzXML format, followed by processing with the XCMS package for R software (developed by the R Core Team, Vienna, Austria) to identify peaks and filter data, and to align the chromatograms. This resulted in a data matrix with retention times, mass-to-charge ratios, and peak intensities, which was exported for metabolomics data analysis described below.

### Statistical and bioinformatic analysis

#### Statistical analysis

Statistical analyses were conducted utilizing the R software. Categorical variables were reported as frequencies (percentages) and compared using the χ^2^ test. Normally distributed continuous variables were presented with their mean and standard deviation (SD), and were evaluated via unpaired Student’s *t*-tests. Non-normally distributed continuous variables were described by their median and interquartile range (IQR), and were analyzed using the Mann–Whitney U-test. Spearman’s rank correlation analysis was employed to examine the associations between microbial, metabolomic, and clinical factors. Univariate linear regression analysis was also performed to determine that specific fecal microbiotas and metabolites were significantly related to eGFR or Hb. Additionally, the random forest algorithm was employed to evaluate the contribution of potential biomarkers in distinguishing CRF-anemia from HC. The accuracy of models for CRF-anemia and HCs was assessed through receiver operating characteristic (ROC) curves generated by pROC with 10-fold cross-validation. All statistical tests were two-tailed, and the *P* value or the false discovery rate (FDR)-adjusted *P* value involved in multiple comparisons correction less than 0.05 was considered statistically significant.

#### Microbiome data analysis

R software was utilized to compare the gut microbiota profiles of CRF patients with anemia and HCs. Species annotation was conducted using Kraken2 with a confidence parameter of 0.2 (37). Following this, we utilized Bracken with its default settings to execute a post-classification Bayesian re-estimation of abundance to ascertain the species-level or genus-level abundance within the metagenomic samples (38). Assessment of alpha diversity was conducted through the computation of the Chao1 richness index and Shannon index. Beta diversity analysis of the fecal microbiota was carried out through PCoA based on the Bray-Curtis distance matrix, and the dissimilarities in community composition among the groups were examined using PERMANOVA, aided by the Adonis function. LEfSe was employed to pinpoint the microbial taxa that distinguished the two groups. The functional annotation was aligned to the protein database (UniRef90) based on DIAMOND by the HUMAnN2 software and KEGG database (39). Differential KEGG pathways were identified based on the Z-scores of individual KO (KEGG ortholog) genes, and their expression levels were compared using the Wilcoxon rank-sum test between the anemia of CRF and HC groups.

#### Metabolomics data analysis

Metabolite identification was initially confirmed based on precise molecular weight (with a molecular weight error of ≤30 ppm). Subsequent annotation included peak picking, retention time correction, and isotope, and adduct annotation was accomplished by matching MS/MS fragmentation patterns with online databases such as HMDB and METLIN (Tables S1 and S2). Functional annotation of metabolites was carried out using KEGG to assign potential biological roles and pathways. We use a comprehensive algorithm that integrates multiple dimensions, such as ppm and fragment response intensity, and compare different parameters with different weights, referring to the libraries to identify substances (40). Metabolites with a relative SD exceeding 30% in the quality control samples were excluded, and the remaining data were subjected to logarithmic transformation. The analysis of metabolic data was also conducted using the R software. The Wilcoxon rank-sum test was used to compare the level of metabolites between the two groups. The interrelationship between gut microbiota composition, fecal metabolites, and serum metabolites was determined by Spearman correlation analysis and the Mantel test.

## Data Availability

Metagenomics raw data have been deposited into NCBI Sequence Read Archive (SRA) database under project accession number PRJNA890567. Serum and fecal metabolomics raw data can be found in MetaboLights under project accession numbers MTBLS12576 and MTBLS6334.

## SUPPLEMENTAL MATERIAL

Supplemental material spectrum.03166-24-s0001.xlsx Tables S1 and S2.

ASM does not own the copyrights to Supplemental Material that may be linked to, or accessed through, an article. The authors have granted ASM a non-exclusive, world-wide license to publish the Supplemental Material files. Please contact the corresponding author directly for reuse.
